# Supplementary material for: Optimising the adult HIV testing services screening tool to predict positivity yield in Zimbabwe, 2022
Source: PLOS Glob Public Health. 2022 Jul 1;2(7):e0000598. doi: 10.1371/journal.pgph.0000598 (PMC10021692; doi:10.1371/journal.pgph.0000598)
Supplement: S1 Text — (DOCX) [file pgph.0000598.s001.docx]

S1 Text_Screening tool evaluation questions

| **Province: ____________________ District: _____________________**  **Facility: ____________________ Completed by: _______________**  **Time start ……..** | |  |
| --- | --- | --- |
| 1 | Sequential number Today’s date Sex Age  M  F  Other |  |
| 2 | Have you ever tested for HIV before?  If yes, what was the result? Positive Negative  If Positive Date of ART initiation: __________________  If negative, when was the most recent test? Month Year |  |
| 3 | \| Marital status (tick appropriate box) \|  \| (month and year) \| \| --- \| --- \| --- \| \| Single \|  \|  \| \| Married to new partner in the last year \|  \|  \| \| Married for longer than a year \|  \|  \| \| Divorced \|  \|  \| \| Separated \|  \|  \| \| Widowed \|  \|  \| \| Cohabiting but not married \|  \|  \|   Do you currently live with a spouse or sex partner? ☐ Yes ☐ No  If yes, in the past year, has your partner sometimes lived away from home for work or other reasons? ☐ Yes ☐ No  Has your sexual partner disclosed their HIV status to you? ☐ Yes ☐ No  Have you disclosed your HIV status to your sexual partner? ☐ Yes ☐ No  If married or has a partner how do you rate your partner’s faithfulness?  Not faithful at all Not sure Very faithful |  |
| 4 | Do you have any sexual partner who tested HIV positive in the last 2 years? ☐ Yes ☐ No |  |
| 5 | In the last 2 years have you ever been:  Physically harmed or threatened by a sexual partner? ☐ Yes ☐ No  Physically forced to have sexual intercourse? ☐ Yes ☐ No |  |
| 6 | In the past 2 years have you  a. Offered/ Given/ Received goods, services, or money in exchange for sex? ☐ Yes ☐ No  b. Shared needles for injecting drugs? ☐ Yes ☐ No  c. Had sex while under the influence of alcohol/drugs? ☐ Yes ☐ No  d. Had sex with someone without a condom, or when the condom has burst? ☐ Yes ☐ No  e. Had sex with more than one sexual partner? ☐ Yes ☐ No |  |
| 7 | Have you been hospitalized or experienced poor health in the past three months? ☐ Yes ☐ No |  |
| 8 | In the past year have you had any of the following symptoms:   - Genital rash ☐ Yes ☐ No - Genital sores ☐ Yes ☐ No - Unusual genital discharge ☐ Yes ☐ No - Persistent or recurrent cough ☐ Yes ☐ No - Significant loss of weight ☐ Yes ☐ No - Recurrent skin rashes ☐ Yes ☐ No |  |
| 9 | On a scale of 1-4 (1-Not at all, 2- Mild, 3- Moderate & 4- severe) how do you rate your HIV risk?  1-Not at all 2-Mild 3-Moderate 4-Severe |  |

**Finish time ……….. Duration ………….. minutes**

**Screening Decision Outcome (Tick applicable)**

- Offered HIV test, Client accepted Code ☐ 0 ☐ 1
- Offered HIV test, Client refused or opted out
