# Supplementary material for: Optimising the adult HIV testing services screening tool to predict positivity yield in Zimbabwe, 2022
Source: PLOS Glob Public Health. 2022 Jul 1;2(7):e0000598. doi: 10.1371/journal.pgph.0000598 (PMC10021692; doi:10.1371/journal.pgph.0000598)
Supplement: S2 Text — (DOCX) [file pgph.0000598.s002.docx]

S2 Text_Screening tool questions for validation

| **Province: ____________________ District: _____________________**  **Facility: ____________________ Completed by: _______________** | | |
| --- | --- | --- |
| Sequential number Today’s date  Format (DMY)    F  M  Sex at birth Age (In completed years) | | |
| 1 | When was the last time you were tested for HIV?  Beyond 12 months  Past 12 months  Past 3 months  Never |  |
| 1a | If previously tested, what was the result?  Positive  Negative  Inconclusive | |
| 2 | If Negative, do you consider yourself to be at risk of HV infection?  No  Yes |  |
| 2a | If Inconclusive |  |
| 2b | If positive, are you currently on ART?  No  Yes  NB: Do not proceed with the tool if client is positive | If No, refer for OI/ART Services |
| 3 | Do you have a sexual partner who tested HIV positive in the last 2 years?  Yes  No |  |
| 4 | Have you experienced poor health in the past 3 months?  *NB: Include TB presumptive symptoms (Productive cough, night sweats, coughing up blood in the past 2 weeks), newly diagnosed clients and weight loss > 10%*  No  Yes |  |
| 5 | Have you experienced any symptoms or signs of an STI such as urethral/vaginal discharge or genital sores?  Yes  No |  |

- Offered HIV test ☐ Client accepted ☐
- HIV Test Results: Negative ☐ Positive ☐ Inconclusive ☐ 1
- Client refused or opted out ☐
