# Supplementary material for: Optimising the adult HIV testing services screening tool to predict positivity yield in Zimbabwe, 2022
Source: PLOS Glob Public Health. 2022 Jul 1;2(7):e0000598. doi: 10.1371/journal.pgph.0000598 (PMC10021692; doi:10.1371/journal.pgph.0000598)
Supplement: S3 Text — (DOCX) [file pgph.0000598.s003.docx]

**S3 Text_Adult HIV Testing Risk Screening Tool Standard Operating Procedure**

**Introduction and Purpose of the Standard Operating Procedure (SOP)**

- This SOP is the implementation guide for the MOHCC adult risk screening tool for HIV testing at facility and community levels for clients (16years and above) before being offered an HIV test.
- The purpose of this SOP is to standardize and guide the implementation of the screening tool across all levels, models and approaches for HTS by all healthcare providers.

**NB**: This screening tool should not be administered to pregnant (Antenatal -ANC) and lactating (Postnatal-PNC), clients on PrEP, PEP, TB and STI confirmed clients.

**Justification for using the HTS screening tool**

The screening tool is designed to assist the tester to decide on which clients need HIV testing, according to their risk profile. This is consistent with the thrust to target HIV testing services and enhance the positivity yield

**Who can administer the Screening Tool**

This guide can be used by any Health service provider involved in HIV testing provision, including Primary Counsellors, Nurses, Doctors, Expert clients (adolescents and adults).

**Interview Guide**

- The interview and the data collected are confidential and should only be used for the intended purpose
- The client is screened in when they indicate that they were last tested **at least 3 months back and respond yes to any of the remaining 4 questions**, If they consent, should be offered HIV testing.
- Clients who are **screened out** should not be tested but may return for a fresh screening session if at risk, at least 3 months from the previous test and not the previous screening date
- Clients should be **screened at least once in** **3 months** since the most frequent testing interval is 3 months according to the retesting algorithm

| No | Questions | Responses |
| --- | --- | --- |
|  | Province | Write the province code in which your site is located |
|  | District | Write the district code in which your site is located |
|  | Facility | Write the facility code that you are administering the tool at |
|  | Sequential number | Record in a numeric format the sequential number of the client in order starting from 1,2,3…etc until the last client in the month ( starting from the first day of the month and ending on the last day of the month) |
|  | Today’s date | Record date of screening in the following format: dd/mm/yr |
|  | Sex at Birth | Regardless of how they identify, ask the client their sex at birth |
|  | Age | Ask the client’s age in completed years and record it in the provided box |
| 1 | When was the last time you were tested for HIV?? | Ask the client last time they had an HIV test done. All clients who were tested less than 3 months are ineligible for testing |
|  | 1a. If previously tested, what was the result? | If the client previously tested for HIV, ask the client to state the results they obtained |
| 2 | If Negative, do you consider yourself to be at risk of HV infection? | Document client self-perception of risk as categorized  Not at all -0, low-1, medium 2 and high.  NB Offer HIV testing for all levels of risk, ie low-1, to high -3 |
|  | 2a. If Inconclusive | If the previous HIV test result was inconclusive and it's now 14 days or more offer HIV testing as per the HTS algorithm. |
|  | 2b. If positive, are you currently on ART? | NB: Do not proceed with the tool if the client is HIV positive Refer the client to OI/ART services. |
| 3 | Do you have a sexual partner who tested HIV positive in the last 2 years? | Find out if the clients had sexual partner/s who tested HIV in the past 2 years. If yes, the client is an index case contact and eligible for HIV testing. |
| 4 | Have you experienced poor health in the past 3 months? | Ask the client if they have been unwell and or admitted to the hospital in the past 3 months and tick the appropriate box. Ill health in the past 3 months includes presumptive TB symptoms such as night sweats, chest pains, productive cough or coughing up blood lasting 2 weeks, loss of appetite, recent diagnosis with TB and unexplained weight loss of > 10%. These are highlighted faintly on the tool as a reminder. Do not influence the client’s perceptions |
| 5 | Have you experienced any symptoms or signs of an STI such as urethral/vaginal discharge or genital sores? | Ask the client if they have had any of the listed symptoms. Include genital itchiness, pain during urination or intercourse, rashes on the genital area, vaginal/urethral discharge, genital/anal sores, blisters or sores in or around the mouth and lower abdominal pain |

**NB:** Proceed to offer HIV testing to all clients that were last tested 3 months or more ago (Question 1), in combination with a yes to any of the subsequent questions (2-5)
