# Supplementary material for: Optimising the adult HIV testing services screening tool to predict positivity yield in Zimbabwe, 2022
Source: PLOS Glob Public Health. 2022 Jul 1;2(7):e0000598. doi: 10.1371/journal.pgph.0000598 (PMC10021692; doi:10.1371/journal.pgph.0000598)
Supplement: Attachment — Submitted filename: Mugauri_Detailed response to reviewer comments.doc [file pgph.0000598.s006.doc]

**Point by Point Response to Editor and Reviewers of Paper** **IJREGI-D-21-00079:**

We thank you all for your time and dedication in going through this paper and we acknowledge the reviews and comments from the editor and reviewers. We provide a point-by-point response to each comment with each response introduced by the word “Author Response” in capital. We have revised the paper accordingly in response to these comments as indicated in our responses below and tracked changes in the manuscript. We hope that the revised paper meets your expectations and may now be suitable for publication in your journal.

Yours sincerely,

Hamufare Mugauri

**Point by Point Response to reviewer comments**

**Reviewer #1**

1. REVIEWER COMMENT: Testing is the gateway to care for people with HIV. Scientifically-proven, cost effective and scalable interventions are recommended by the CDC and WHO as a way to improve outcomes in HIV prevention especially in regions where HIV prevalence is high. The screening tool described in this study meets this threshold.

   While the study uses a cross sectional survey, having a comparison group would have provided a stronger link between this tool and the positivity yield and whether the change observed would be directly attributed to the use of this tool. The change described in the conclusion of this study did not take place with a comparison group who did not receive the same intervention. As it is, we can not confidently conclude that the change we see is due to the screening tool alone. Therefore, the results we have here may hold but with a recommendation for future research to include a comparison group. Robustness of the findings was demonstrated by performing sensitivity analysis and this is a strength for the study.

   Overall, the study objective is reflected in the summarized findings and the authors report a balanced record of the analyzed results.

AUTHOR RESPONSE

Thank you for the compliment on the soundness of our study, the robustness of the findings and for acknowledging that it presents a balanced record of the analysed results. This is in line with our thinking too.

Our study was cross-sectional. It recruited all clients that reported at the sampled facilities where all eligible participants had a screening tool administered and their responses matched with the HIV test result during analysis. This was to facilitate measuring sensitivity, specificity, predictive value positive (PVP) and negative (PVN) and the number needed to test (NNT). Because of the nature of the study and the analysis done, we could not recruit an independent comparison group. Instead, our comparison was among recruited participants in respect of how they responded to each question (Ye/No) against the outcome of their HIV test. Our comparisons were between clients who responded with a Yes, against those who responded with a No, considered in the context of their HIV test result. Therefore, whilst we acknowledge the value of a comparison group in research analysis, this was not possible in our study. It is possible, though, to conduct a study that evaluates the effectiveness of a screening tool where one group receives the tool whilst the control group doesn’t and then compare the outcome of the HIV test results, but this is not what we did.

**Reviewer #2**

1. REVIEWER COMMENT**:** This is a very interesting and important piece of work that will help many countries to adapt and tailor the screening tool for HIV. I would like to congratulate the authors on this work

AUTHOR RESPONSE: Thank you for the compliment. We concur that this work will serve as part of the pioneering work to assist other countries to adapt and tailor their tools as well as evaluations and validations following the model that we used.

2. REVIEWER COMMENT**:** However, there are major issues with the way this paper has been written and designed:
   1. The authors need to define upfront what the tools consisted of. After many paragraphs in the intro, it is said that it was a questionnaire. The tool should be defined and briefly described giving the overall view of the screening tool
   AUTHOR RESPONSE: Thank you for this suggestion. We have adjusted by introducing the developed screening tool for evaluation, which was also the data documentation sheet **(Annexe 1)** in the introduction. Line 71-78 of the introduction has been edited to read; “*The concept of screening for eligibility for an HIV test was introduced in 2019, but was deficient of a clearly defined criteria for screening clients in and out of testing, neither were the questions assessed for their performance. This therefore resulted in this screening approach reducing the testing volumes from 3 million in 2018, to 2.3 million in 2019..*” However, we considered explaining the screening questions that formed the tool which was evaluated under the methods section since this was the data collection tool. Line 150-155 now reads “*Data were collected from 1 to 30 April 2021 for the initial phase (evaluation) using a structured data collection form, which was the developed draft screening tool* ***(S1 Annex).*** *The questions were extrapolated from commonly used risk profiling questions both locally and regionally, resulting in a total of 13 questions being evaluated. These were printed and used as data collection tools where health workers documented the response to questions and also the HIV test results.”*
3. REVIEWER COMMENT: 2. The authors discuss that the tool was adapted and many other variables were added and subcategories were made, but this needs to be again described briefly much before the analysis subsection as the actual work is not clear before the analysis section and this can get difficult for the reader to understand.

AUTHOR RESPONSE: Thank you for pointing this out. We have revised the entire data collection section to indicate that we started by evaluating a total of 13 questions, which were a combination of the existing screening questions and the added questions to make the exercise robust. We further referenced the documents which are attached as Annexes. We then described the second phase (validation), stating the criteria of selecting the 5 questions which made it, referenced the tool as annexe 3 and provided the SOP which guided the process. This is reflected in 153-174.

4. REVIEWER COMMENT**:** 3. in line 189 it is mentions, "Various combinations were tested....", this is again getting complicated as it is not clear what combinations are the authors talking about? The questions in the tool or the categories in the answers? Or the addition/deletion of questions in the tool to attain high sensitivity?

AUTHOR RESPONSE: Thank you for this observation. We have revised the statement to make it clearer in line 211-213 to read; *“We explored combining different questions until the best output results of sensitivity were achieved, according to how participants had responded to these questions”.*

5. REVIEWER COMMENT**:** 4. The discussion needs to succinctly mention overall changes that were made and how it impacted.

AUTHOR RESPONSE: Thank you for pointing this out. We have added, in line 494-498 the following; “*The developed screening tool proposes a shift in how the screening process for HIV testing is conducted. This is a shift from determining eligibility for testing through a yes response from any of the 7 previous questions whose properties were unknown, to utilizing a defined criteria for determining eligibility for HIV testing using a 5-question screening tool whose properties are now documented.*”. We believe that the impact of using the developed screening tool will be felt when the tool in now in use and we reflect on whether the attributes documented during the evaluation and validation translates to what will happen at national scale.

6. REVIEWER COMMENT: 5. The discussion also needs to discuss how it increased positivity rates- were they more close to the actual HIV incidence in these regions? As the regions were randomly selected, the %positivity can be compared to these regions selected rather than the national incidence. This will help understand the impact of this modified screening tool better.

AUTHOR RESPONSE: Thank you for pointing this out. Ewe have revised line 446-456 to read; *“First, HIV positivity yield remained comparable during the time of the study and before the evaluation, 7.52% during evaluation and 7.98% during validation* ***(Table1&2),*** *against 7.83% (Programmatic data for the same sites for March 2021). This was an observation of concern. Considering that a screening tool was already being utilized to determine eligibility for testing, with clients being screened in and others screened out, it was expected that during the evaluation, the positivity yield would have declined since all clients were being tested, regardless of the screening outcome. This finding influenced us to hypothesize that either the screening tool was not being routinely utilized to determine eligibility for testing, or the tool was being used and yet not effective to correctly screen in and out, which is also supported by the fact that the tool had not been evaluated”.* The process of evaluating and validating the screening tool would not increase positivity rates, but rather decline it because everyone was being tested to measure the attributes of the questions by matching responses to HIV test results. The implementation of the tool is expected to improve positivity yield, which would require a follow-up study to evaluate if the attributes obtained during evaluation and validation will be observed on large scale implementation.

7. REVIEWER COMMENT**:** It is very difficult to understand the overall message that the authors are trying to tell. It is only later in the paper that the reader can understand what the actual message is.
   Such an important piece of work needs more finesse in writing so that the message does not get diluted.
8. AUTHOR RESPONSE: Thank you for this observation. We have refined the entire manuscript to bring out the key message of developing a screening tool to guide testers in determining eligibility for testing. Further, in the conclusion, we added in line 526-528 that: “This tool provides a scientific way of determining eligibility for testing which is critical in targeting HIV testing to increase positivity yield and expedite meeting espoused HIV testing milestones”.
